# Designer Self-Assembling Peptide Nanofiber Scaffolds for Adult Mouse Neural Stem Cell 3-Dimensional Cultures

**DOI:** 10.1371/journal.pone.0000119

**Published:** 2006-12-27

**Authors:** Fabrizio Gelain, Daniele Bottai, Angleo Vescovi, Shuguang Zhang

**Affiliations:** 1 Center for Biomedical Engineering, Massachusetts Institute of Technology, Cambridge, Massachusetts, United States of America; 2 Stem Cell Research Institute, Department of Biological and Technological Research, Fondazione Centro San Raffaele del Monte Tabor, Milan, Italy; 3 Bioscience and Biotechnology Department, University of Milan-Bicocca, Milan, Italy; 4 Department of Medicine, Surgery and Dentistry, Faculty of Medicine, University of Milan, Milan, Italy; Baylor College of Medicine, United States of America

## Abstract

Biomedical researchers have become increasingly aware of the limitations of conventional 2-dimensional tissue cell culture systems, including coated Petri dishes, multi-well plates and slides, to fully address many critical issues in cell biology, cancer biology and neurobiology, such as the 3-D microenvironment, 3-D gradient diffusion, 3-D cell migration and 3-D cell-cell contact interactions. In order to fully understand how cells behave in the 3-D body, it is important to develop a well-controlled 3-D cell culture system where every single ingredient is known. Here we report the development of a 3-D cell culture system using a designer peptide nanofiber scaffold with mouse adult neural stem cells. We attached several functional motifs, including cell adhesion, differentiation and bone marrow homing motifs, to a self-assembling peptide RADA16 (Ac-RADARADARADARADA-COHN2). These functionalized peptides undergo self-assembly into a nanofiber structure similar to Matrigel. During cell culture, the cells were fully embedded in the 3-D environment of the scaffold. Two of the peptide scaffolds containing bone marrow homing motifs significantly enhanced the neural cell survival without extra soluble growth and neurotrophic factors to the routine cell culture media. In these designer scaffolds, the cell populations with β-Tubulin^+^, GFAP^+^ and Nestin^+^ markers are similar to those found in cell populations cultured on Matrigel. The gene expression profiling array experiments showed selective gene expression, possibly involved in neural stem cell adhesion and differentiation. Because the synthetic peptides are intrinsically pure and a number of desired function cellular motifs are easy to incorporate, these designer peptide nanofiber scaffolds provide a promising controlled 3-D culture system for diverse tissue cells, and are useful as well for general molecular and cell biology.

## Introduction

Nearly all tissue cells are embedded in a 3-dimensional (3-D) microenvironment in the body. However, almost all tissue cells have been studied in 2-D Petri dishes, 2-D multi-well plates or 2-D glass slides coated with various substrata. The architecture of the *in situ* environment of a cell in a living organism is 3-D, where cells are surrounded by other cells as well as many extracellular ligands, including many types of collagens, laminin, and other matrix proteins. The normal three-dimensional environment of cells comprises a complex network of extracellular matrix nanoscale fibers with nanopores that create various local microenvironments. These environments not only allow attachments between cells and the basal membrane, but also allow access to oxygen, hormones and nutrients, as well as removal of waste products.

The movements of cells in the 3-D environment of a living organism typically follow a chemical signal or molecular gradient, which is crucial for organism development. It is known that cells isolated directly from higher organisms frequently alter their metabolism and gene expression patterns in 2-D culture. Cells growing in a 2-D environment may significantly reduce production of particular extracellular matrix proteins and often undergo morphological changes, for instance, an increase in spreading.

Conventional 2-D cell cultures are unlike *in vivo* systems where cellular communication, transport of oxygen and nutrients, removal of wastes and cellular metabolism take place in a 3-D environment.

Attempts have been made to culture cells in 3-D using synthetic polymers and their copolymers [1]. However, many processed synthetic polymers consist of microfibers ∼10–50 micrometers in diameter, which are similar in size to most cells (∼5–10 micrometers in diameter). Thus, cells attached to microfibers are still in a two-dimensional environment with a curvature dependent on the diameter of the microfibers. Furthermore, the pores (∼10–200 micrometers) between the microfibers are often ∼1,000–10,000 times larger than the size of biomolecules, which have sizes just a few nanometers, including small molecular hormones, proteins, growth and other factors, which consequently can quickly diffuse away. For a true 3-D environment, a scaffold's fibers and pores must be substantially smaller than the cells. Although synthetic biopolymer microfiber scaffolds have been studied for over 30 years to mimic *in vivo* 3D microenvironment, concerns about their degradation products and chemicals involved in their synthesis are still important issues requiring further improvements.

Animal derived biomaterials such as collagen gels, laminin, poly-glycosaminoglycans and materials from basement membranes, including Matrigel™ have also widely been used in cell cultures [2]–[9]. While they are representative of the correct nanolength scale, they often contain residual growth factors, undefined constituents or non-quantified substances [2]–[6]. This not only makes it difficult to conduct well-controlled studies with these materials, but also poses problems if such scaffolds are ever to be used for growing tissues for human therapies.

An ideal 3-D cell culture system should be fabricated from a synthetic biological material with defined constituents. We previously reported the discovery of a self-assembling peptide system, made from natural amino acids, that can undergo spontaneous assembly into nanofiber scaffolds, ∼10 nm in fiber diameter with pores between 5–200 nm [10]–[12]. These peptides have been chemically produced in large quantity using standard solid phase synthesis method and purified to homogeneity. They have not only been used for the study of cell attachment, survival and proliferation but also to incorporate other motifs [11]–[16], and inject into animals [17]–[19]. These self-assembling peptides form nanofibers that can be controlled at physiological pH by altering salt concentration [10]–[11]. Because the self-assembled nanofibers are several thousand times thinner than synthetic polymer microfibers and cells, thus it is believed that the peptide nanofibers surround cells in a manner similar to the natural extracellular matrix. However a systematic study of different motifs and an examination of how their nanofiber scaffolds interact with cells in details have not been carried out.

Here we report the use of designer peptide nanofiber scaffolds to produce 3-D cultures for the study of mouse adult neural stem cells. We synthesized 18 different peptides that directly incorporate various functional motifs with the self-assembling peptide RADA16. These motifs include sequences shown to promote cell adhesion, differentiation and bone marrow homing activities. These functionalized peptides self-assemble into nanofiber scaffolds where cells can be fully embedded by the scaffold in 3-D. Without addition of soluble growth factors and neurotrophic factors, two of these scaffolds functionalized with bone marrow homing motifs [20] not only significantly enhanced survival of the neural stem cells, but also promoted differentiation towards cells expressing neuronal and glial markers. This is the first example suggesting that designer peptide scaffolds alone without additional extra growth factors could influence neural stem cell differentiation towards neural and glial phenotypes.

## Results

### Designer peptide synthesis

This class of peptide is short and can be readily molecular-designed from knowledge gained in the literature, we designed and produced the functionalized self-assembling peptides. The self-assembling peptide RADA16 was appended with motifs from various types of collagen [21]–[22], laminin [23], fibrin [24], fibronectin [25]–[26], osteopontin and osteogenic peptides [26]–[27], bone marrow homing peptides (BMHP) [20] and myelo-regulatory peptides [28]–[29]. The incorporation of these functional motifs did not prevent the designer peptides from forming well-ordered nanofibers using SEM examination.

**Figure 1:**
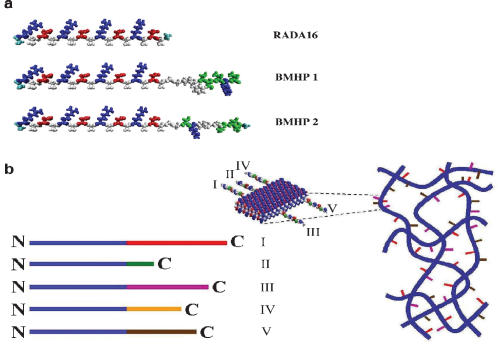
Molecular and schematic models of the designer peptides and of the scaffolds. a) Molecular models of RADA16, RADA16-Bone Marrow Homing Peptide 1 (BMHP1) and RADA16-Bone Marrow Homing Peptide 2 (BMHP2). RADA16 is an alternating16-residue peptide with basic arginine (blue), hydrophobic alanine (white) and aspartic acid (red). These peptides self-assemble once exposed to physiological pH solutions or salt. The alanines of the RADA16 providing hydrophobic interaction are on one side of the peptide, and the arginines and aspartates form complementary ionic bonds on the other. The BMHP1 and BMHP2 motifs were directly extended from RADA16 with two glycine spacers and are composed of a lysine (blue), serine and threonine (green) and different hydrophobic (white) residues. Neutral polar residues are drawn in green. b) Schematic models of several different functional motifs (different colored bars) could be extended from RADA16 (blue bars) in order to design different peptides (I, II, III, IV and V). They can be combined in different ratios. A schematic model of a self-assembling nanofiber scaffold with combinatorial motifs carrying different biological functions is shown.

These designer peptides were synthesized by extending the C-termini of the self-assembling peptide RADA16 (Ac-RADARADARADARADA-COHN2) (Fig. 1A) to include the functional motifs. Two glycine residues as a spacer linker were used for flexibility between the assembling portion of the peptides and the functional motifs. A schematic illustration is shown in Figure 1B. Blue lines represent the self-assembling peptide main sequence, and the red, green, purple, yellow and brown lines represent various functional peptide motifs. The functional motifs are located on the C-termini, as a result of solid phase peptide synthesis that starts from the C-termini, in order to reduce sequence errors during synthesis.

For this study, we selected functional sequences found in neural cell adhesion motifs, collagen fragments and bone marrow differentiating and homing peptides as well as myelo-peptides and osteogenic growth peptides. Particularly, we tested RGD-based sequences from fibronectin (RGDS) and from collagen VI (PRGDSGYRGDS) that have been found to improve the sprouting of hippocampal mouse neurons, laminin derived motifs (YIGSR, IKVAV, PDSGR) already adopted as promoters of neurite regeneration *in vivo* experiments [23]–[24] and neurite sprouting *in vitro* with other self-assembling peptides [30]; a bioregulatory mediator peptide from the family of myelo-peptides (GFLGFPT) suggested to influence differentiation in bone marrow and peripheral blood cells in humans [28]–[29]. We also tested two other motifs, BMHP1 (SKPPGTSS) and BMHP2 (PFSSTKT), which belong to a family of peptides (bone marrow homing peptides) rich in K, P, F, S, and T, which have been shown to home into bone marrow *in vivo* [20]. A detailed description and the full-length sequences are provided in Table 1.

**Table 1:**
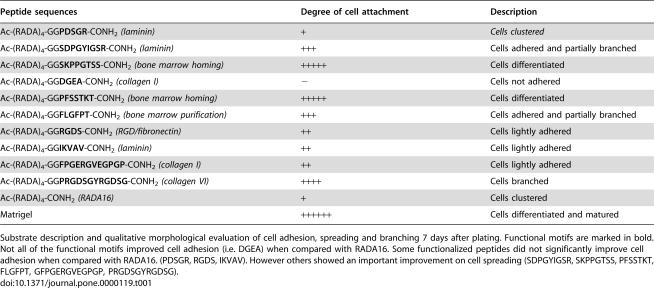
Designer self-assembling peptides used in this study.

Peptide sequences	Degree of cell attachment	Description
Ac-(RADA)_4_-GG**PDSGR**-CONH_2_ *(laminin)*	+	*Cells clustered*
Ac-(RADA)_4_-GG**SDPGYIGSR**-CONH_2_ *(laminin)*	+++	Cells adhered and partially branched
Ac-(RADA)_4_-GG**SKPPGTSS**-CONH_2_ *(bone marrow homing)*	+++++	Cells differentiated
Ac-(RADA)_4_-GG**DGEA**-CONH_2_ *(collagen I)*	−	Cells not adhered
Ac-(RADA)_4_-GG**PFSSTKT**-CONH_2_ *(bone marrow homing)*	+++++	Cells differentiated
Ac-(RADA)_4_-GG**FLGFPT**-CONH_2_ *(bone marrow purification)*	+++	Cells adhered and partially branched
Ac-(RADA)_4_-GG**RGDS**-CONH_2_ *(RGD/fibronectin)*	++	Cells lightly adhered
Ac-(RADA)_4_-GG**IKVAV**-CONH_2_ *(laminin)*	++	Cells lightly adhered
Ac-(RADA)_4_-GG**FPGERGVEGPGP**-CONH_2_ *(collagen I)*	++	Cells lightly adhered
Ac-(RADA)_4_-GG**PRGDSGYRGDSG**-CONH_2_ *(collagen VI)*	++++	Cells branched
Ac-(RADA)_4_-CONH_2_ *(RADA16)*	+	Cells clustered
Matrigel	++++++	Cells differentiated and matured

Substrate description and qualitative morphological evaluation of cell adhesion, spreading and branching 7 days after plating. Functional motifs are marked in bold. Not all of the functional motifs improved cell adhesion (i.e. DGEA) when compared with RADA16. Some functionalized peptides did not significantly improve cell adhesion when compared with RADA16. (PDSGR, RGDS, IKVAV). However others showed an important improvement on cell spreading (SDPGYIGSR, SKPPGTSS, PFSSTKT, FLGFPT, GFPGERGVEGPGP, PRGDSGYRGDSG).

All of the designer peptides are soluble in aqueous solution and self-assemble into nanofiber scaffolds upon exposure to cell culture medium, PBS, and in neutral pH solutions.

The peptides of RADA16, RADA16-BMHP1 and RADA16-BMHP2 are modeled using the van der Waals representation and colored using the residue type method (Fig. 1). The original main sequence of alternating basic (blue), hydrophobic (white) and acid (red) residues is the RADA16 sequence necessary for the self-assembling property of the functionalized scaffold. In this manner, a simplified designer peptide scaffold model is proposed with different functional motifs that could be easily incorporated (Fig. 1B).

### Designer peptide nanofiber scaffolds and cells embedded in them

**Figure 2:**
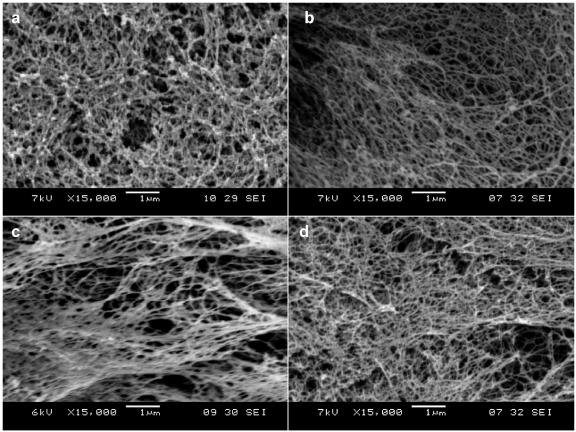
SEM images of Matrigel and various designer peptide nanofiber scaffolds. a) Matrigel, b) RADA16, c) RADA16-BMHP1, d) RADA16-BMHP2 nanofiber scaffolds assembled in PBS solutions. Matrigel nanostructures are comparable in size to nanofibers found after self-assembly of the designer peptides. Clusters and aggregates of the unidentified naturally derived proteins in Matrigel (a) are absent in the pure peptide scaffolds shown in (b), (c) and (d). The interwoven nanofibers are ∼10 nm in diameter in each of the peptide scaffolds with ∼5–200 nm pores. The appended functional motifs did not prevent peptide self-assembly.

Upon incorporation of functional motifs to the RADA16 peptide, there was concern that the appended peptide motifs would inhibit the self-assembling peptide nanofiber formation. To address the concern, we used scanning electron microscopy (SEM) to examine the nanofiber structure of these designer peptides. We show here the expected nanofibers of the pure RADA16 and of the 100% functionalized peptides after self-assembly both in PBS and in cell culture medium (Fig. 2).

First we examined the ultra-structures of the commonly used Matrigel. The SEM images of Matrigel revealed ordered nanofibers with nanopores and some aggregates, which presumably are proteins (Fig. 2A). On the other hand, the designer peptide nanofibers appear to be smooth with dimensions and pore size comparable to Matrigel, and consistent with previous reports [11]–[12]. There are remarkable structural similarity of nanofibers of the pure RADA16 scaffold (Fig. 2B), the peptide scaffolds with addition of the functional motifs BMHP1 (Fig. 2C) and BMHP2 (Fig. 2D). Similar nanofibers with nanopores also formed with other functionalized designer peptides scaffolds (data not shown). These findings open the door for combinatorial tests of many different functional motifs in the future experiments.

**Figure 3:**
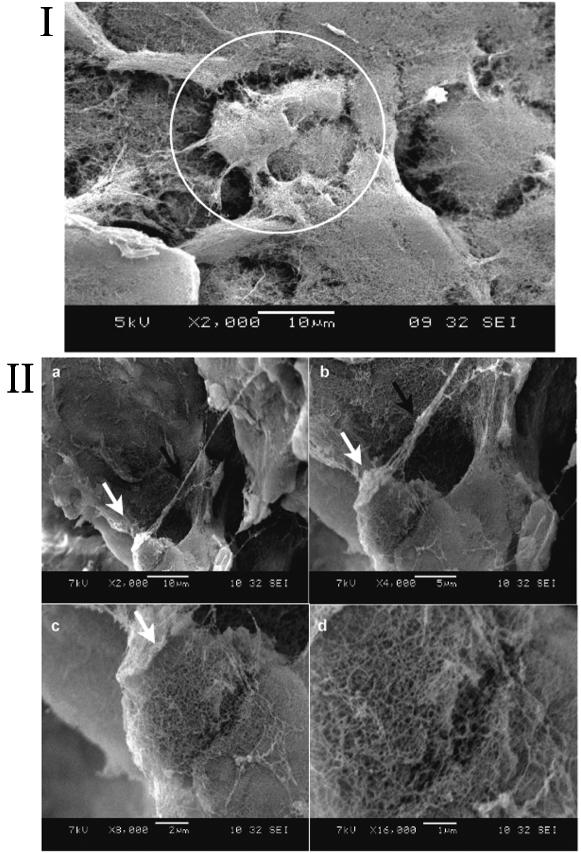
SEM images of adult mouse neural stem cells (NSC) embedded in designer peptide nanofiber scaffold RADA16-BMHP1 (1% v/w) after 14 day *in vitro* cultures. I) Cluster of three visible NSCs (white circle) embedded in 3-D self-assembling RADA16-BMHP1. II) A single cell at different magnification with extended processes embedded in the scaffold is shown (a–c). White arrows point to the image areas enlarged in the consecutive pictures. d) High-magnification picture focusing on the interface between the nanofiber scaffold and the round shaped cell body. The black arrow in (b) points to a cellular process. Cells and processes are thus embedded in the self-assembling peptide nanofiber scaffold in a true 3-D environment, which may likely promote cell adhesions in 3-D similar to the natural cellular environment. Adult mouse neural stem cells have been cultured and could be differentiated *in vitro* for several weeks. The scale bars are shown on each image.

In order to examine cell-scaffold interactions, we carried out experiments using SEM and found that cells were truly embedded in the nanofiber scaffolds (Fig. 3). The cell bodies appear to be fully embedded in the designer peptide nanofiber scaffolds. The nanofiber scale is similar to an ECM-like environment. These images provide the evidence that self-assembling peptide nanofiber scaffolds embed cells in a true 3-D microenvironment. The arrow in Figure 3 points to a single location at increasing magnifications. As can been seen from the images, cells intimately interact with the surrounding scaffold (Fig. 3). Such interaction may not be likely on 2-D coated surfaces. These images also suggest that cells may remodel the surrounding peptide nanofibers for intimate biomechanical interactions.

### Cell viability and survival

**Figure 4:**
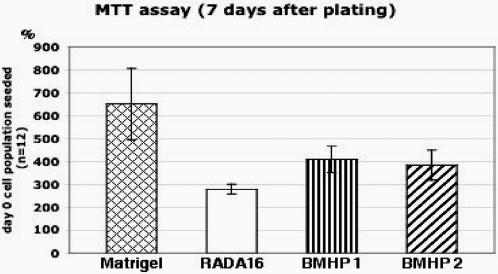
MTT cell proliferation assays of adult mouse neural stem cells after 7-day culture. Cells were seeded on Matrigel or peptide scaffolds. The results are expressed as cell % increases from the seeding population on first day. BMHP1 and BMHP2 peptide scaffolds allow for higher cell proliferation in comparison to RADA16 peptide scaffold (t = −7.28 and t = −5.28 for respectively RADA16 vs. RADA16-BMHP1 and RADA16 vs. RADA16-BMHP2 with p<0.0001% in both cases). Not surprisingly, Matrigel containing various unknown quantity of growth factors showed considerable cell population increase. Similar increases of total cell populations were confirmed for 14-day cultures (not shown). The cell proliferation using the designer peptide scaffolds could be further improved from addition of soluble neurotrophic factors.

We quantitatively tested the total viable cell population after 7-days of cell culture using the standard MTT assay. Results in Figure 4 are expressed as a percentage of the corresponding initial population (see methods for details). Cells proliferated in all of the mentioned substrates to more than 2.5 times the initial cell population seeded.

The seeded mouse neural stem cells exhibit higher levels of attachment on some of the tested scaffolds, with the best viability and survival found on Matrigel, RADA16-BMHP1 and RADA16-BMHP2. Some of the other peptide scaffolds are less effective in maintaining cell viability and attachment (Table 1) when compared with the RADA16 scaffold, and thus they are not discussed further here.

It is important to emphasize that in these 3-D cultures, no extra soluble growth factors (except for those in the cell media, see methods for details) and adhesion proteins were added. This is in sharp contrast to maintaining cell viability with a variety of soluble growth factors in conventional 2-D cell cultures.

Not surprisingly, Matrigel, a cell culture extract containing a variety of adhesion proteins and growth factors, showed the higher level of cell survival and viability, twice the living cell population in comparison with the pure RADA16 scaffold (Fig. 4). Of the functionalized peptides, RADA16-BMHP1 and RADA16-BMHP2 scaffolds showed ∼25% increase in cell population when compared with the RADA16 scaffold (significant differences with P<0.0001 for both RADA16 vs. RADA16-BMHP1 and RADA16 *vs.* RADA16-BMHP2). Similar results for these substrates were observed after 14 days of culture as well (results not shown).

### Cell Differentiation from the functionalize motifs

**Figure 5:**
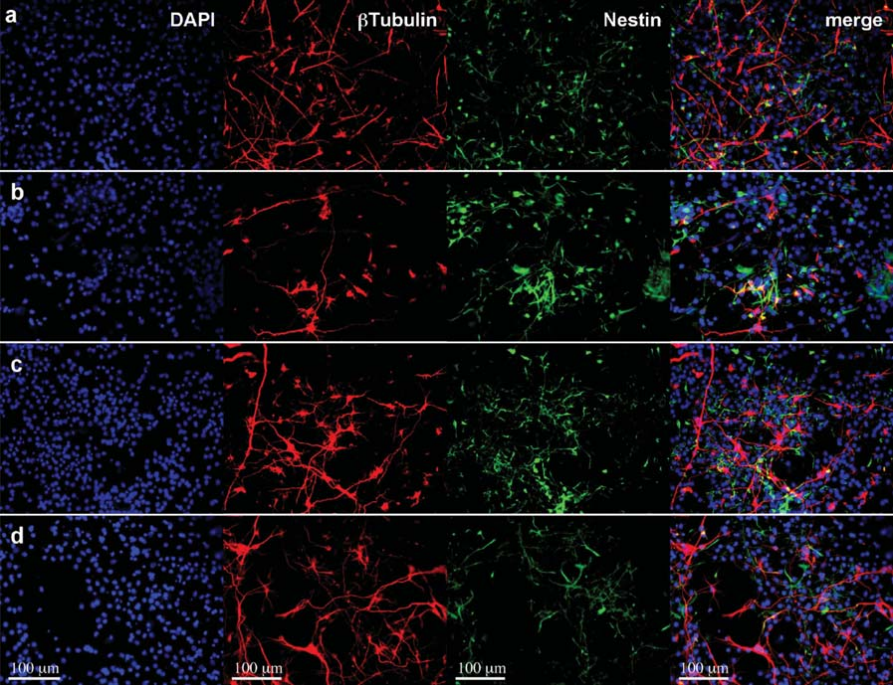
Images of neural stem cells in differentiation assays. a–d) Differentiating adult mouse neural stem cells cultured for 7 days *in vitro* on (a) 1% Matrigel, (b) RADA16 peptide scaffold; (c) RADA16-BMHP1 and (d) RADA16-BMHP2, 1% peptide scaffolds were examined with staining assays for DAPI (cell nuclei in blue), β-Tubulin^+^ (neurons in red), and Nestin^+^ (neural progenitors in green). β-Tubulin^+^ cells on BMHP1 and BMHP2 showed increased branching in comparison with RADA16 scaffold. These appearances are comparable with neurons on Matrigel coated wells. Nestin^+^ and β-Tubulin^+^ signals show negligible cross-reaction (MERGE images).

The mouse neural stem cell differentiation was evaluated 7 days after seeding. Cells were stained with two well-described markers, beta-tubulin for neurons (red) and nestin for neural progenitors (green). Several scaffolds were used: Matrigel (Fig. 5a), RADA16 peptide (Fig. 5b), RADA16-BMHP1 (Fig. 5c) and RADA16-BMHP2 (Fig. 5d). The cells in the two designer peptide scaffolds exhibited neuronal branching similar to that found in the Matrigel (Fig. 5a). The neuronal processes on the functionalized scaffolds appear significantly extended when compared with the processes on the RADA16 scaffold (Fig. 5b). Staining the cells with GFAP marker for astrocytes resulted in similar findings (see Supporting Figure S2): while astrocytes with a predominantly bipolar glial morphology were seen on the RADA16 scaffold, astrocytes with a multipolar glial morphology were observed on the RADA16-BMHP1, RADA16-BMHP2 and Matrigel scaffolds.

**Figure 6:**
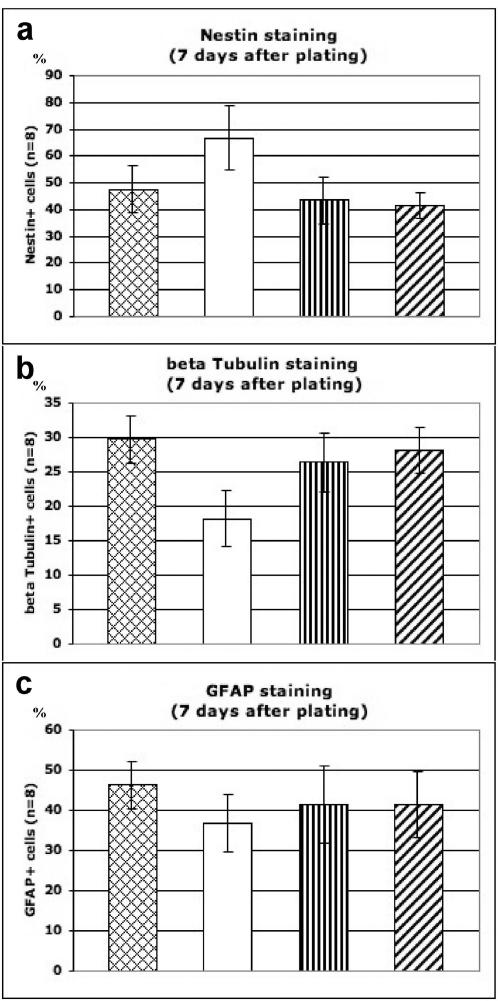
Quantitative cell differentiation assays. The adult mouse neural stem cells were stained with various markers 7 days after culturing on Matrigel (positive control), RADA16, RADA16-BMHP1 and RADA16-BMHP2 peptide scaffolds. Values are expressed as averages±STD. a) % Nestin^+^ progenitor cell was reduced on the cultures of Matrigel, RADA16-BMHP1 and RADA16-BMHP2 peptide scaffolds, suggesting cell differentiation. b) % β-Tubulin^+^ cell was similar for Matrigel culture, RADA16-BMHP1 and RADA16-BMHP2 peptide scaffolds, suggesting neuronal differentiation. c) % GFAP^+^ cell was slightly higher in Matrigel culture, RADA16-BMHP1 and RADA16-BMHP2 peptide scaffolds, suggesting the ability of glial cells to adapt to a wide range of substrates.

Quantitative results are expressed as a percentage of the total cell population (DAPI cell nuclei staining) (Fig. 6). Of all functionalized scaffolds tested (results for other scaffolds not shown), RADA16-BMHP1 and RADA16-BMHP2 exhibited the lowest percentage of positive cells for the progenitor marker Nestin (43.4%±8.9 for BMHP1; 41.5%±4.8 for BMHP2) (Fig. 6a). Conversely, these two functionalized scaffolds exhibited the highest percentage of positive cells for the neuronal marker β-Tubulin (26.4%±4.2 for BMHP1; 28.2%±3.4 for BMHP2) (Fig. 6c) and for the astrocyte marker GFAP (41.4%±9.5 for BMHP1; 41.3%±8.2 for BMHP2) (Fig. 6d). These values are similar to those found with Matrigel (47.5%±8.7 Nestin positive cells; 29.7%±3.5 β-Tubulin positive cells; 46.4%±6 GFAP positive cells).

Our experimental results show that for RADA16 scaffold more cells remain in the undifferentiated state and fewer neurons are found. However, the scaffolds appended with simple and short functional motifs play a previously unsuspected role that influences cell behaviors. These pure designer peptide scaffolds with defined functional motifs influenced the mouse neural stem cell differentiation nearly on par with heterogeneous Matrigel.

### Gene expression analysis after differentiation from the functionalize motifs

We carried out a quantitative Real-Time-PCR using an RT^2^ *Profiler*™ plate. The plate contained a set of primers for 84 genes expressed for the extracellular matrix and adhesion molecules. We compared the gene expression profiles of the cells that were cultured in Matrigel, RADA16 and RADA16-BMHP1. The analysis was carried out in two separate preparations for each functionalized motif in triplicate for each sample. Namely, we repeated on three different plates the PCR for the same cDNA and we repeated the experiment twice.

Among the expression, 33 of the 84 genes were detected (we considered only the genes that were detected at least in two out of three PCR experiments); 30 in the Matrigel sample, 12 in RADA16 sample, 5 in the RADA16-BMHP1 (Table 2).

**Table 2:**
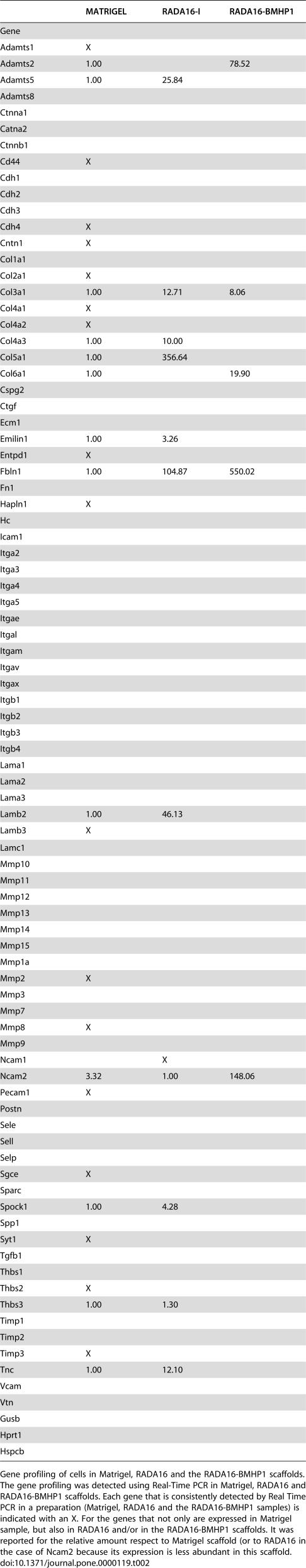
Gene expression profiling in three nano scaffolds.

	MATRIGEL	RADA16-I	RADA16-BMHP1
Gene			
Adamts1	X		
Adamts2	1.00		78.52
Adamts5	1.00	25.84	
Adamts8			
Ctnna1			
Catna2			
Ctnnb1			
Cd44	X		
Cdh1			
Cdh2			
Cdh3			
Cdh4	X		
Cntn1	X		
Col1a1			
Col2a1	X		
Col3a1	1.00	12.71	8.06
Col4a1	X		
Col4a2	X		
Col4a3	1.00	10.00	
Col5a1	1.00	356.64	
Col6a1	1.00		19.90
Cspg2			
Ctgf			
Ecm1			
Emilin1	1.00	3.26	
Entpd1	X		
Fbln1	1.00	104.87	550.02
Fn1			
Hapln1	X		
Hc			
Icam1			
Itga2			
Itga3			
Itga4			
Itga5			
Itgae			
Itgal			
Itgam			
Itgav			
Itgax			
Itgb1			
Itgb2			
Itgb3			
Itgb4			
Lama1			
Lama2			
Lama3			
Lamb2	1.00	46.13	
Lamb3	X		
Lamc1			
Mmp10			
Mmp11			
Mmp12			
Mmp13			
Mmp14			
Mmp15			
Mmp1a			
Mmp2	X		
Mmp3			
Mmp7			
Mmp8	X		
Mmp9			
Ncam1		X	
Ncam2	3.32	1.00	148.06
Pecam1	X		
Postn			
Sele			
Sell			
Selp			
Sgce	X		
Sparc			
Spock1	1.00	4.28	
Spp1			
Syt1	X		
Tgfb1			
Thbs1			
Thbs2	X		
Thbs3	1.00	1.30	
Timp1			
Timp2			
Timp3	X		
Tnc	1.00	12.10	
Vcam			
Vtn			
Gusb			
Hprt1			
Hspcb			

Gene profiling of cells in Matrigel, RADA16 and the RADA16-BMHP1 scaffolds. The gene profiling was detected using Real-Time PCR in Matrigel, RADA16 and RADA16-BMHP1 scaffolds. Each gene that is consistently detected by Real Time PCR in a preparation (Matrigel, RADA16 and the RADA16-BMHP1 samples) is indicated with an X. For the genes that not only are expressed in Matrigel sample, but also in RADA16 and/or in the RADA16-BMHP1 scaffolds. It was reported for the relative amount respect to Matrigel scaffold (or to RADA16 in the case of Ncam2 because its expression is less abundant in this scaffold.

Three of the 84 genes were detectable in all three samples: Fbln 1 (Fibulin 1), procollagen type III α1 and neural cell adhesion molecule 2 and fibulin. Seven genes were detected in Matrigel and RADA16 including a disintegrin-like and metalloprotease (reprolysin type) with thrombospondin type 1 motif 5, elastin microfibril interfacer 1, protocollagen type IV α3, protocollagen type V α3, thrombospondin 3, tenascin C, Laminin β2. Two genes were present at the same time in the samples Matrigel and RADA16-BMHP1: A disintegrin-like and metalloprotease (reprolysin type) with thrombospondin type 1 motif 2, procollagen type VI α1. 15 genes were measurable only in the Matrigel sample: disintegrin-like and metalloprotease (reprolysin type) with thrombospondin type 1 motif 1, cadherin 4, contactin 1, procollagen type II α1, procollagen type IV α1, procollagen type IV α2, ectonucleoside triphosphate diphosphohydrolase 1, hyaluronan and proteoglycan link protein 1, laminin β3, matrix metalloproteinase 2, platelet/endothelial cell adhesion molecule, thrombospondin 2, tissue inhibitor of metalloproteinase 3. Cadherin 1 and proteoglycan 1 were only detectable in the RADA16-BMHP2 samples; whereas neural cell adhesion molecule 1 was detectable only in mRNA isolated from cells cultured in RADA16.

**Figure 7:**
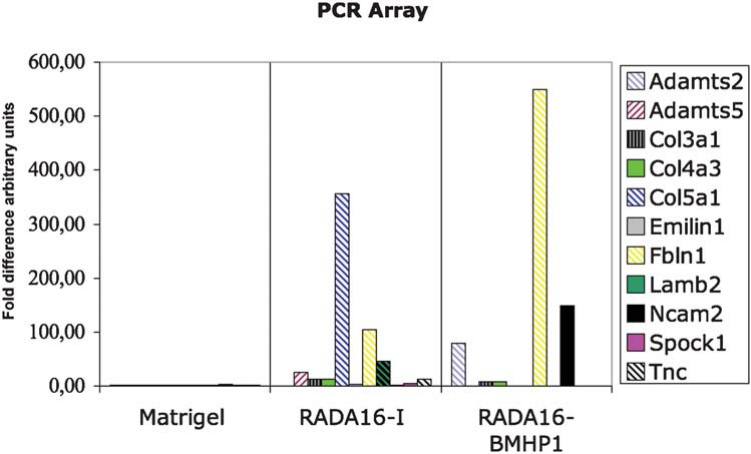
Comparisons of the gene expression profiling. Adamts2–5: disintegrin-like and metalloprotease (reprolysin type) with thrombospondin type 1 motif, 2 or 5; Col3a1: Procollagen, type III, alpha 1; Col4a3: Procollagen, type IV, alpha 3; Col5a1: Procollagen, type V, alpha 1; Col5a1: Procollagen, type V, alpha 1; Emilin1: Elastin microfibril interfacer 1; Fbln1: Fibulin 1; Lamb2: Laminin, beta 2; Ncam2: Neural cell adhesion molecule 2; Spock1: Sparc/osteonectin, cwcv and kazal-like domains; Tnc: Tenascin C.

For the genes that are detected at least in two different samples we performed a comparison calculating the fold differences assigning to the sample that had the lower level the value 1. Fibulin 1 has low expression level in the Matrigel sample whereas in RADA16, RADA16-BMHP1 the levels of expression relative to Matrigel are 105, 550 higher respectively. Neural cell adhesion molecule 2 is highly expressed in RADA16-BMHP1. The gene expression profiling is showed in Fig. 7 and in Table 2

## Discussion

### Synthetic polymer microfibers and self-assembling peptide nanofibers

Most of the synthetic polymer biomaterials currently used in tissue cell cultures and regenerative medicine usually have microfiber structures [1], [31]–[34]. Since the size of most cells (5–10 µ) are similar or smaller to these microfibers (∼10–100 µ), upon attachment to the microfibers the cells still exhibit a 2-D topology with a curvature depending on the diameter of the microfibers.

Polymeric materials are often functionalized to promote desired biological activities through chemical reactions or coating. Because of their micro-scale sizes, their mechanical strength usually prevents further material structural adaptations from the forces exerted by cells during their adhesion, migration and maturation processes. Thus, although these microfibers provide an artificial extracellular environment, they are still far from the natural nanoscale ECM.

For the encapsulation of labile bioactive substances and living cells, physically cross-linked nanofiber scaffolds are of great interest, especially the scaffold formation occurs under mild physiological conditions without any organic solvent processes. There is a need for process scaffolds in aqueous environment with a desired pH range, temperature or specific catalysts that could control their microstructure properties.

We here show that peptide nanofiber scaffolds formed from a variety of functionalized designer self-assembling peptides are likely a promising alternative. This is a new class of biologically inspired materials that mimics the 3-D nanostructure of the extracellular matrix and that of Matrigel. Although we here only used mouse neural stem cells to carry out this study of these designer scaffolds, many cell types have been studied using the pure RADA16 peptide scaffold [12]–[19] and more are under the way.

### Designer peptide nanofibers with functional motif scaffolds

The self-assembling peptides with incorporated functional motifs can be produced with high purity at a reasonable cost commercially. Thus, this method can be readily adopted for wide spread uses including study how cell interact with their local- and micro-environments, cell migrations in 3-D, tumor and cancer cells interactions with the normal cells, cell process and axon extension, cell based drug test assays and other diverse applications.

We have produced different designer peptides from a variety of functional motifs with different lengths. Although their nanofiber structures appear to be indistinguishable from the RADA16 scaffold, the appended functional motifs significantly influenced cell behaviors.

Using the designer self-assembling peptide nanofiber system, every ingredient of the scaffold can now be defined and combined with various functionalities including the soluble factors. This is in sharp contrast with a 2-D Petri dish where cells attach and spread only on the surface; now the cells reside in a 3-D environment where the extracellular matrix receptors on the cell membranes can bind to the functional ligands appended to the peptide scaffolds. It is possible that higher tissue architectures with multiple cell types, rather than monolayers, could be constructed using these designer 3-D self-assembling peptide nanofiber scaffolds.

Even if only a fraction of functionalized motifs on the 3-D scaffold are in all probability available for cell receptor binding, cells likely receive more external stimuli than when in contact with coated 2-D Petri dishes or polymer microfibers. In a 2-D environment, where only part of the cell body is in contact with the surface, receptor clustering at the attachment site may be induced; on the other hand, the receptors for growth factors, cytokines, nutrients and other signals are on the sides that expose directly with the culture media. In the 3-D environment, the functional motifs on the nanofiber scaffold surround the whole cell body in all dimensions and the factors may form a gradient in 3-D nanoporous microenvironment.

Our results show high levels of differentiation of mouse neural stem cells toward both neuronal and glial phenotypes in the designer scaffolds *in vitro* in well-controlled conditions. These results are similar to those with Matrigel, a natural extract considered as the most effective and standard cell-free substrate for neural stem cell culture and differentiation. The designer peptide scaffolds with functional motifs not only significantly improve mouse neural stem cell survival, but also enhance their differentiation when compared to the non-functionalized self-assembling sequence RADA16.

In addition to the laminin-derived sequence previously studied with self-assembling peptides [30], this study evaluates common fibronectin and collagen derived sequences as well. In our search for additional functional motifs, we found that a class of bone marrow homing peptides BMHP [20] could be one of the most promising family candidates for stimulating NSC adhesion and differentiation. BMHP1 is homologous to N-terminal CD84, a cell surface protein expressed by lymphocyte progenitors [35]. While BMHP2 belongs to the same family as BMHP1, its biochemical function still remains to be studied [20]. It is believed that these designer scaffolds containing such motifs may be useful not only for stem cell research, but also for future neural tissue regeneration.

Bone marrow is one source of adult stem cells that can differentiate into endothelial, muscle and connective tissue and neuronal cells. It is likely that bone marrow cells, as a source for adult stem cells, have some of the same differentiating pathways and adhesion receptors as NSCs [36].

In this study, we showed that the addition of motifs (up to 12 additional residues) to the self-assembling peptide RADA16 did not inhibit self-assembling properties and nanofiber formations. This study suggests a new class of designer self-assembling peptides for 3-D cell biology studies.

### Different Gene expressions in Matrigel and designer peptide nanofiber scaffold

Some of the gene screened by PCR RT^2^ *Profiler*™ (APM-013-2) resulted in a different gene profile expression when compared with Matrigel. Within these genes, Fibulin 1 has rather low expression level in the Matrigel scaffold, whereas in RADA16 and RADA16-BMHP1 the levels of expression relative to Matrigel are 105 and 550 respectively in the PCR-array. Fibulins are secreted glycoproteins defined by the presence of two structural modules, namely a collection of repeated epidermal growth factor (EGF)-like domains and a unique C-terminal Fibulin-type module. Some fibulin genes encode several protein products through alternative splicing, a process that involves the differential processing of exon–intron junctions to yield new transcript variants. For example, the alternative splicing of Fibulin-1 transcripts results in four separate variants termed -1A to -1D, which differ at the C-terminus of the protein. Fibulins exhibit an extensive array of protein–protein interactions, particularly with other extracellular matrix (ECM) proteins. Indeed, it is thought that fibulins act as intramolecular bridges within the ECM, connecting various supramolecular structures, and mediate certain cell signaling events [37]. A tumor-suppressive role has been suggested for fibulin-1. The over-expression of fibulin-1D in fibrosarcoma-derived cells reduced anchorage-independent growth *in vitro*, in addition to delayed tumor formation *in vivo* [38], moreover, both ectopically expressed fibulin-1D and purified fibulin-1 protein have been shown to inhibit the cell adhesion, spreading, motility and invasion of a range of human tumor-cell lines *in vitro* [39]. It is not clear which splice variant is expressed in our 3-D culture system, perhaps it may be plausible that in presence of RADA16 or RADA16-BMHP1, the high levels of fibulin may play a role in tumor suppression. It remains to be further experiments to test the idea.

Another gene which expression seems to change drastically when the differentiation is performed in presence of RADA16 or RADA16-BMHP1 instead of Matrigel is Laminin-β2. Laminins are a family of extracellular matrix proteins. They are trimers formed by an α-, β-, and γ subunit. During development, laminins 2 and 8 are the major trimers expressed by Schwann cells, with laminin 8 expressed at higher levels than in the adult [40]–[41]. Low levels of β2 mRNA have been described in neurons and satellite cells [41]. Laminins contribute to basal assembly by interacting with other matrix components such as fibulins.

The contemporarily increase of mRNA expression of Fibulin 1 and Laminin-ß2 in peptide scaffold differentiation is particularly intriguing and maybe due to a similar or common pathway of activation of the gene expression by means of the interaction between the cells and the synthetic scaffold.

From the MTT assay we expected that the cells cultured in Matrigel express genes coded for the proteins required for ECM interaction in Matrigel, indeed the real time PCR assay showed that among the 84 genes analyzed, 30 were detected in the Matrigel scaffold, but only few in the designer peptide scaffolds. Some of the genes detected in the designer peptide scaffolds were expressed at a much higher level than in the Matrigel, probably to compensate the limited interaction possibilities. The complete panel of the gene profiling is reported in Table 2. It also shows the relative difference in gene expressions that are not only expressed in the Matrigel, but also in RADA16 and/or in the RADA16-BMHP1 scaffolds.

### The advantages of designer peptide nanofiber scaffolds *vs.* other scaffolds

The advantage of using the designer peptide nanofiber scaffolds is several folds. 1) One can readily modify the designer peptides at the single amino acid level at will, inexpensively and quickly. This level of modification is impossible with Matrigel and other polymer scaffolds. 2) Unlike Matrigel, which contains unknown ingredients and quality that varies from batch to batch, the designer self-assembling peptide scaffolds belong to a class of synthetic biological scaffolds that contains pure components and every ingredient is completely defined. 3) Because these designer peptide scaffolds are pure with known motifs, it can be used to study controlled gene expression or cell signaling process. Thus these new designer nanofiber scaffolds proved to be promising tools to study, cell signal pathways in a selective way not possible with any substrates including Matrigel and collagen gels that result in confusing cell signaling activation. 4) The initiation of the self-assembly process is through change of ionic strength at the physiological conditions without temperature influence. This is again unlike collagen gels, for which the gelation is through change of temperature that can sometimes induce unknown biological process including cold or heat shocks. 5) These scaffolds provide the opportunity to incorporate a number of different functional motifs and their combinations to study cell behavior in a well-defined ECM-analog microenvironment, not only without any chemical cross-link reactions but also fully bio-reabsorbable scaffolds.

### Designer peptide scaffold for regenerative medicine

Beyond 3-D cell culture, since the building blocks of this class of designer peptide scaffold are made of pure natural L-amino acids, RADA16, unlike most of the other synthetic microfibers, has been shown not to elicit detectable immune response or inflammatory reactions in animals [17]–[19], and the degraded products can be reused by the body. Therefore, this class of scaffold may be useful as a bio-reabsorbable scaffold for neural repair and neuroengineering to alleviate and treat a number of neuro-trauma and neuro-degeneration diseases.

Quantitative biology requires *in vitro* culture systems that more authentically represent the cellular environment in a living organism. In doing so, *in vitro* experimentation can become truly more predictive of *in vivo* systems.

## Methods

### Designer peptide synthesis and scaffold preparation

All peptide sequences used in this work were dissolved in distilled sterile water (GIBCO) at a final concentration of 1% (v/w) (10 mg/ml) and sonicated for 30 min.

Cell viability and differentiation assay tests were conducted by pouring aqueous solution of functionalized sapeptide (30 µl per well) so as to evenly cover the bottom surface of each well (approximately 30 µm gel layer thickness) of 96 multi-well plates (BD Biosciences). In the case of SEM imaging both for Matrigel and the sapeptides, the total amount of biomaterial was reduced to 10 µl. The experimental protocol included control tests conducted with non-functionalized RADA16 (negative control) and Matrigel coating (positive control). RADA16 and other functionalized sapeptides: poured 30 µl/well of a 1% (w/v) distilled sterile water solution, followed by slow addition of 200 µl/well of basal medium. Allowed to self-assemble at +37°C for 30 minutes and rinsed once with control medium to wash away any residual acid residues remaining from peptide synthesis. Matrigel GF-reduced (from EHS sarcoma, BD Biosciences): diluted 1∶100 in basal medium, poured at 100 µl/well, 30′ incubation at 37°C, then rinsed with basal medium. In the case of SEM imaging, no dilution was adopted in order to guarantee the necessary stiffness for a 3-dimensional scaffold.

### Cell cultures and seeding

Neural precursor cultures were established and expanded as described in the supplementary methods.

In the case of adhesion and differentiation tests, cell seeding (at a concentration of 2–8×10^4^ cells/cm^2^) was performed two days after the last mechanical dissociation in order to seed the maximum percentage of stem cells. Cells were seeded on the top-surface of each assembled scaffold, where they were able to settle into the nanofiber matrices. Over time cells penetrate the self-assembled layer (see Supporting Fig. S1).

In the case of SEM imaging, cells were acutely mixed with 8 µl of aqueous gel solution at a final concentration of 5–8×10^3^ cells/μl in a total final volume of 10 µl per each sample. Self-assembling was then initiated by adding basal medium slowly and placing seeded scaffolds mounted on copper grids (Ted Pella Inc.) at +37°C for 30 minutes. Cells were thus already embedded in the matrices.

For both adhesion and differentiation tests and SEM imaging, cells were cultured with basal medium supplemented with βFGF (10 ng/ml), added to enhance neuronal progeny differentiation. After 3 days, the medium was shifted to a medium containing Leukemia Inhibitory Factor (LIF, Chemicon) (20 ng/ml) and Brain Derived Neurotrophic Factor (BDNF, Peprotech) (20 ng/ml) to pursue the neuronal and glial population maturation in NSC progeny [42]. Cells were fed every three days with the same fresh culture medium.

### Cell proliferation assay

To assess the viability of NSCs seeded on scaffolds made of various peptides, a quantitative method, MTT assay (Sigma), was used (see supplementary methods). Four independent experiments comprising three replicates each were performed. For this viability test the direct proportional linearity between the optical density and the viability/metabolic activity of the cell populations was assessed from verifying the linearity of 5 different standard curves at 6 increasing cell concentrations, ranging from 5×10^3^ to 5×10^5^ cells/well. Results are expressed as percent increase in cell population from the population seeded on day one.

### Immunocytochemistry

Neuronal and glial differentiation was assessed by double and single immunostaining with lineage-specific antibodies: anti-Nestin (1∶150, Chemicon) for progenitor cells, rabbit anti-β-Tubulin (1∶500, Covance) for neurons, mouse anti-Glial Fibrillary Acidic Protein (1∶200, Chemicon) for astrocytes. Primary antibodies were then stained with secondary ALEXA 488 goat anti-mouse (1∶1000 Molecular Probes) and CY3 AffiniPure F(ab')2 Anti-Rabbit IgG antibodies (1∶100 Jackson Immuno Research). Cell nuclei were counterstained with DAPI (Molecular Probes). The samples were then examined by inverted fluorescence microscope. Quantitative analyses were performed by counting 100–300 cells for each of 10 non-overlapping (and randomly chosen) fields. Four independent experiments comprising two replicates each were performed.

### SEM sample preparation and imaging

After seeding NSCs within the self-assembled scaffolds as previously described (cell seeding section), cells were cultured for 7 and 14 days in NSC basal medium. The peptide matrices were prepared for SEM as described in the supplementary methods and examined using a JOEL JSM 6060 SEM at 2,000–100,000× magnification, 6KV acceleration voltage, 29–32 spot size, and 12 mm electronic working distance.

### RNA isolation and DNAse1 treatment

Two independent preparations were used per each functional motif scaffolds. Total RNA was isolated in accordance to the manufacturer's instructions. To remove the chromosomal DNA, the samples were incubated with DNase I (2 U/1 µg of RNA) (Ambion, Austin, TX) at 37°C for 60 min in a total volume of 50 µl. DNase-treated RNA was extracted again with phenol-chloroform-isoamyl-alcohol and precipitated in ethanol.

### cDNA synthesis

Synthesis of single-strand cDNA was carried out using 300 ng of random hexamers (Invitrogen, San Diego, CA), and 5 µg of RNA in a total volume of 11 µl at 65°C for 10 min and chilled on ice. Then, 8 µl of reaction mix (1 first strand buffer, 5 mM dithiothreitol (DTT), 2.5 mM of dNTPs, 20 U RNAout™ (Invitrogen, San Diego, CA) and 200 U M-MLV Reverse Transcriptase III (Invitrogen, San Diego, CA), was added. The reaction was incubated at 25°C for 5 min, at 42°C for 1 hour and at 70°C for 10 min.

### SYBR® Green I real-time PCR

Real-time PCR was performed in a MJ Chromo 4 using Brilliant® SYBR® Green QPCR Master Mix (Stratagene, La Jolla, CA). Amplification was performed in a total volume of 25 µl containing Brilliant® SYBR® Green QPCR Master Mix 1× and template cDNA. After a cycle of 95°C for 10 min, the reactions were cycled 40 times under the following parameters: 95°C for 30 sec, then 40 s, 56°C for 30 sec, 72°C for 1 min. At the end of the PCR, the temperature was increased from 60°C to 95°C at a rate of 2°C/min, and the fluorescence was measured every 15 sec to construct the melting curve. A non-template control (NTC) was run with every assay, and all determinations were performed in triplicate in two separated experiments. The analysis was performed using an RT^2^ *Profiler*™ (APM-013-2) PCR Array from SuperArray Bioscience Corporation.

## Supporting Information

Figure S1 Confocal image (LEICA) of neural stem cells 3 weeks after plating initially seeded on the top surface of BMHP1 scaffold. Living cells were labeled in green with Live/Dead assay kit from Molecular Probes and cell nuclei were labeled in blue with Hoechst 33342. Cells penetrated the self-assembled matrix underneath them and formed a 3D cellular network.
(4.62 MB TIF)

Figure S2 Images of astrocytes cell assays. (a) Staining for DAPI (cell nuclei in blue) and GFAP+ cells (astrocytes in green) of adult mouse neural stem cells seeded after 7 days in vitro on 1% Matrigel coated wells, (b) RADA16; (c) RADA16-BMH1 and (d) RADA16-BMH2 peptide scaffolds. Astrocytes cells were detected in the all peptide scaffolds tested.
(9.65 MB TIF)
