# Supplementary figures and images for: Designer Self-Assembling Peptide Nanofiber Scaffolds for Adult Mouse Neural Stem Cell 3-Dimensional Cultures
Source: PLoS One. 2006 Dec 27;1(1):e119. doi: 10.1371/journal.pone.0000119 (PMC1762423; doi:10.1371/journal.pone.0000119)

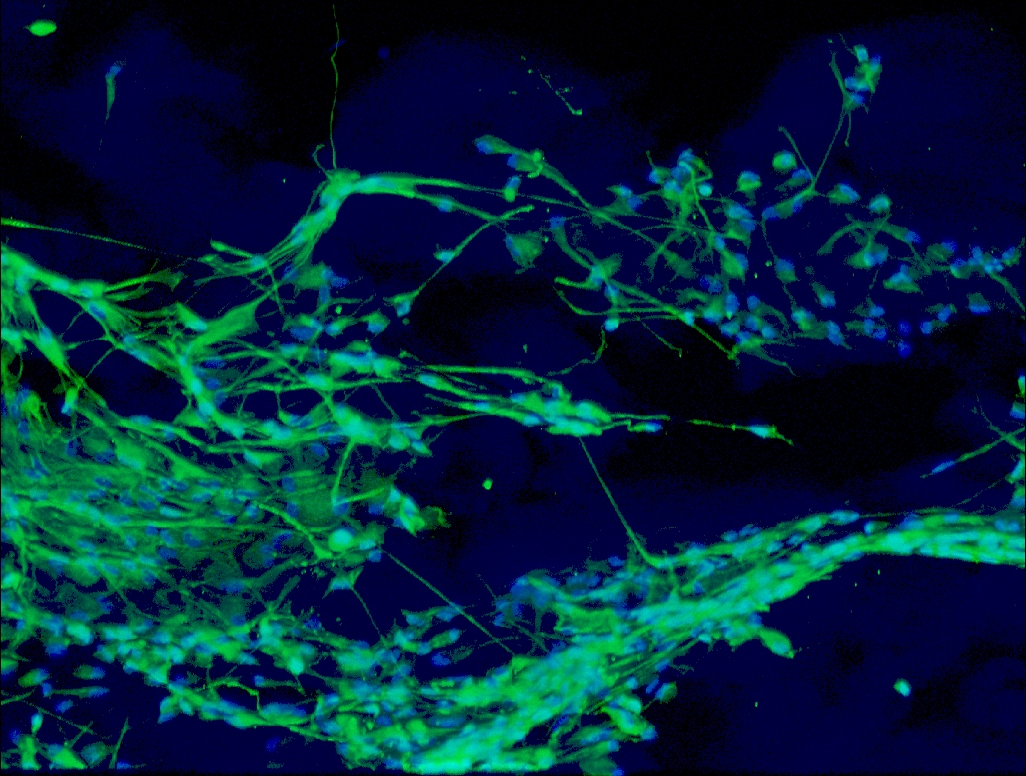

Supplement: Figure S1 — Confocal image (LEICA) of neural stem cells 3 weeks after plating initially seeded on the top surface of BMHP1 scaffold. Living cells were labeled in green with Live/Dead assay kit from Molecular Probes and cell nuclei were labeled in blue with Hoechst 33342. Cells penetrated the self-assembled matrix underneath them and formed a 3D cellular network. (4.62 MB TIF) [file pone.0000119.s001.tif]

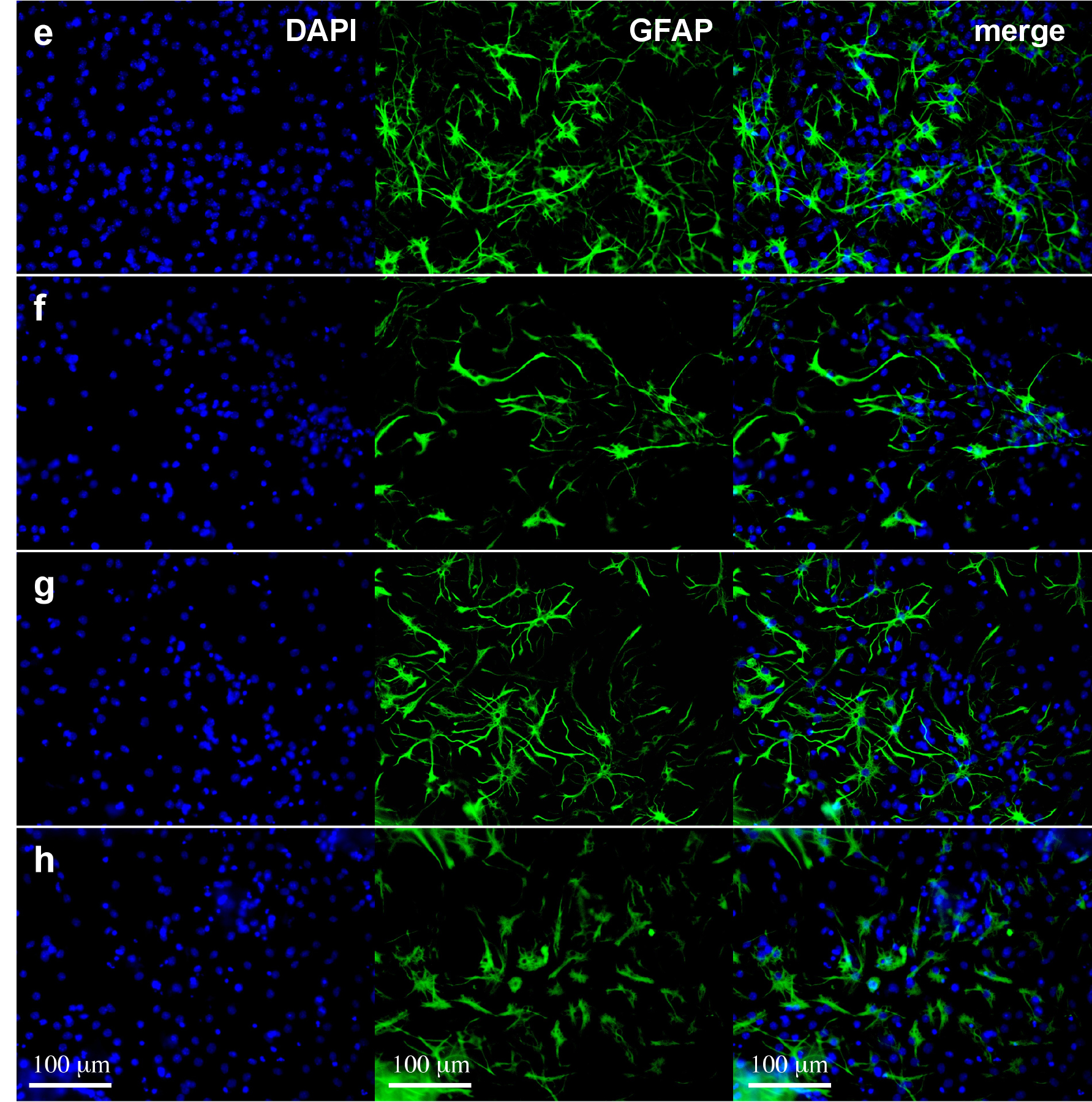

Supplement: Figure S2 — Images of astrocytes cell assays. (a) Staining for DAPI (cell nuclei in blue) and GFAP+ cells (astrocytes in green) of adult mouse neural stem cells seeded after 7 days in vitro on 1% Matrigel coated wells, (b) RADA16; (c) RADA16-BMH1 and (d) RADA16-BMH2 peptide scaffolds. Astrocytes cells were detected in the all peptide scaffolds tested. (9.65 MB TIF) [file pone.0000119.s002.tif]
